# Involvement of pentraxin‐3 in the development of hypertension but not left ventricular hypertrophy in male spontaneously hypertensive rats

**DOI:** 10.14814/phy2.70086

**Published:** 2024-10-16

**Authors:** Siluleko A. Mkhize, Ashmeetha Manilall, Lebogang Mokotedi, Sule Gunter, Frederic S. Michel

**Affiliations:** ^1^ Integrated Molecular Physiology Research Initiative, Faculty of Health Sciences, School of Physiology University of the Witwatersrand Johannesburg South Africa; ^2^ Department of Physiology, School of Medicine Sefako Makgatho Health Sciences University Pretoria South Africa; ^3^ Department of Health Sciences and Medicine Bond University Gold Coast Queensland Australia

**Keywords:** hypertension, inflammation, left ventricular hypertrophy, pentraxin‐3, vascular cell adhesion molecule‐1

## Abstract

Hypertension drives the development of concentric left ventricular hypertrophy (LVH). However, the relative contribution of pentraxin‐3 (PTX‐3), a novel marker for inflammatory cardiovascular disease, in the hypertrophic response to pressure overload has not been adequately elucidated. We investigated the role of PTX‐3 in the development of LVH in spontaneously hypertensive rats (SHR), untreated and treated with either captopril (an ACE inhibitor) or hydralazine (a non‐specific vasodilator). Three‐month‐old SHR received either 20 mg/kg/day hydralazine (SHR + H, *n* = 6), 40 mg/kg/day captopril (SHR + C, *n* = 6), or plain gelatine cubes (untreated SHR, *n* = 7) orally for 4 months. Wistar Kyoto rats (WKY, *n* = 7) were used as the normotensive controls. Blood pressure (BP) was measured using the tail‐cuff method. Cardiac geometry and function were determined using M‐mode echocardiography. Circulating concentrations of inflammatory markers were measured in plasma by ELISA. LV fibrosis and cardiomyocyte width were assessed by histology. Relative mRNA expression of *PTX‐3* was determined in the LV by RT‐PCR. Untreated SHR exhibited greater systolic BP and relative wall thickness (RWT) compared to WKY. Captopril and hydralazine normalized BP but only captopril reversed RWT in SHR. Circulating PTX‐3 and VCAM‐1 levels were elevated in untreated SHR and reduced with captopril and hydralazine. Circulating PTX‐3 was positively associated with systolic BP but lacked independent relations with indices of LVH. LV relative mRNA expression of *PTX‐3* was similar between the groups. PTX‐3 may not be involved in the development of LVH in SHR, but plausibly reflects the localized inflammatory milieu associated with hypertension.

## 1 INTRODUCTION

In the setting of hypertension, the development of concentric left ventricular hypertrophy (LVH) has been subject to profound interest, grounded in the principles of La Place's law (Lorell & Carabello, 2000). As the heart contends with heightened afterload, the left ventricle (LV) undergoes a dynamic process of adaptation in order to maintain optimal wall stress (Lorell & Carabello, 2000). In addition to La Place's law, the interplay of molecular pathways in the development of concentric LVH is intricate and complex (Samak et al., 2016). In this regard, the use of an angiotensin‐converting enzyme (ACE) inhibitor, that decreases the systemic and tissue activation of the renin‐angiotensin‐aldosterone system (RAAS), has been shown to have a greater beneficial effect on the development of concentric LVH beyond its anti‐hypertensive effect (Chen et al., 2020). Conversely, hydralazine, a non‐specific vasodilator that may activate the sympathetic nervous system, presents with nuanced results on the development of concentric LVH in hypertensive models. While lowering blood pressure (BP), hydralazine may lack efficacy in attenuating the development of concentric LVH (Katholi & Couri, 2011). Taken together, these data demonstrate the importance of targeting the molecular pathways in addition to the control of hypertension in order to reduce concentric LVH.

Amongst other molecular pathways activated during hypertension, inflammation may significantly influence the development of concentric LVH (Masiha et al., 2013). Pentraxin‐3 (PTX‐3), an acute‐phase protein released in response to the activation of the innate immune system (Inoue et al., 2012), has gained attention for its potential role in cardiovascular pathology (Ristagno et al., 2019). PTX‐3 is a member of the pentraxin superfamily of proteins, which also includes the well‐known C‐reactive protein (CRP). PTX‐3 is a long pentraxin, structurally and functionally distinct from short pentraxins like CRP and serum amyloid P (SAP) (Li et al., 2024). In particular, PTX‐3, which functions as a local inflammatory mediator, has been shown to contribute to tissue repair and remodeling (Doni et al., 2015; Ristagno et al., 2019). In hypertensive patients, circulating PTX‐3 concentration is increased and negatively correlates with flow‐mediated dilatation, an index of endothelial function (Alp et al., 2019). However, the role of PTX‐3 in the development of endothelial dysfunction and vascular remodeling remains controversial (Carrizzo et al., 2015; Zlibut et al., 2019). The discrepant data are mirrored by in vitro studies wherein PTX‐3 has been shown to have differing effects on the vasculature by modulating angiogenesis (Camozzi et al., 2006; Leali et al., 2012; Rusnati et al., 2004), impairing regenerative response in vessels (O'Neill et al., 2016), and influencing the progression of athrogenesis (Gustin et al., 2008; Liu et al., 2014). In respect of concentric LVH in response to increased afterload, results are also controversial.

In a murine model of pressure overload achieved by transverse aortic constriction, LV hypertrophic changes, assessed as heart mass normalized to body mass and interventricular septal thickness, were attenuated by a deficiency in the expression of *PTX‐3* while an overexpression increased these changes (Suzuki et al., 2013). In contrast, the administration of recombinant PTX‐3 protein in spontaneously hypertensive rats (SHR) with heart failure ameliorated the LV remodeling and systolic dysfunction associated with hypertension (Chen, Zhuang, et al., 2021). In addition to the lack of consensus, both studies used supraphysiological concentration of PTX‐3 leaving a gap on the effect of physiological level of PTX‐3 (circulating or myocardial) on the development of concentric LVH in hypertension.

Understanding the precise roles and regulatory mechanisms of PTX‐3 may lay the foundations for novel interventions aimed at mitigating inflammation‐driven cardiac and vascular damage. In this regard, studies elucidating the context‐specific functions of PTX‐3 and exploring its crosstalk with other inflammatory mediators in the setting of hypertension are lacking. We therefore aimed to determine the role of circulating PTX‐3 concentration or LV relative mRNA expression of *PTX‐3* in the development of concentric LVH in SHR using two anti‐hypertensive therapies with differential effects on the development of concentric hypertrophy.

## 2 MATERIALS AND METHODS

### 2.1 Animals and housing

The present study was approved by the Animal Research Ethics Committee of the University of the Witwatersrand (clearance no. 2021/03/03C), conducted in accordance with the National Institutes of Health Guide for the Care and Use of Laboratory Animals, and conforms to the Animal Research: Reporting of In vivo Experiments (ARRIVE) guidelines (Percie du Sert et al., 2020). The animals were housed individually under conditions of controlled ambient temperature with a light–dark cycle of 12‐h with ad libitum access to drinking water and standard laboratory animal chow (Labchef, Johannesburg, South Africa).

### 2.2 Study design

**FIGURE 1:**
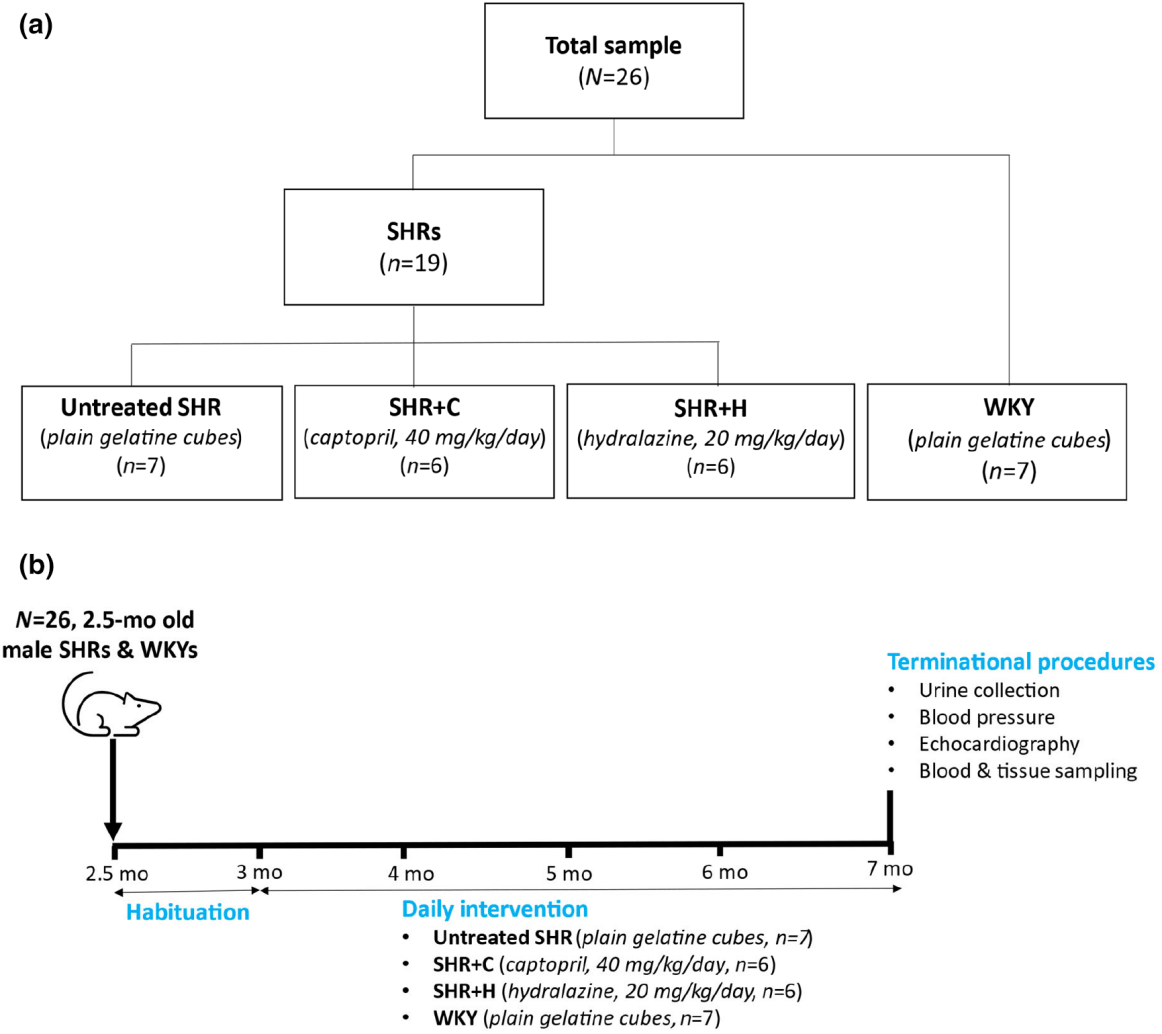
(a) A diagrammatic illustration of the study design. (b) A diagrammatic illustration of the sequence and timeline of the experimental procedures.

Twenty‐six (*n* = 26), 3‐month‐old male rats consisting of spontaneously hypertensive rats (SHR, *n* = 19, RRID:RGD_10043828) and normotensive Wistar‐Kyoto rats (WKY, *n* = 7, RRID:RGD_61119) were sourced and housed at the Wits Research Animal Facility (WRAF). In SHR, the hypertrophic response to pressure overload is sexually dimorphic (Al‐Gburi et al., 2017). It is for this reason that we utilized male rats in the present study. For a period of 2 weeks, the rats were allowed to acclimatize to the housing environment and habituated to consumption of plain gelatine cubes. Thereafter, the SHR were randomly assigned to three groups. The first group (SHR, *n* = 7) received plain gelatine cubes. The second group (SHR + H, *n* = 6) received hydralazine at a dose of 20 mg/kg/day in a gelatine cube (Li et al., 2020). The third group (SHR + C, *n* = 6) received captopril at a dose of 40 mg/kg/day in a gelatine cube (Ernsberger et al., 2007). The normotensive WKY (*n* = 7) also received plain gelatine cubes. Figure 1a shows a diagrammatic illustration of the study design. The selected doses have been previously shown to elicit antihypertensive effects in SHR. The antihypertensive therapies or the plain gelatine cubes were given for 4 months. To ensure complete oral uptake of the drugs, bovril was added to improve palatability of the gelatine cubes. The gelatine cubes were eaten immediately by the rats. In the literature, a sample size of six to eight rats per group was adequate to show statistical differences in this model (Wilson et al., 2017). Figure 1b shows a diagrammatic illustration of the sequence and timeline of the experimental procedures.

### 2.3 Non‐invasive blood pressure measurement

For each animal, BP measurement was performed once a week for the last 4 weeks prior to termination. BP was measured using a tail‐cuff technique (Mokotedi et al., 2019) which employs non‐invasive BP (NIBP) amplifiers (Biopac systems, Santa Barbara, USA). A built‐in pump automatically inflates the tail cuff to occlude the vessel in the tail of a rat and then slowly deflates when the inflation point is reached, providing a linear drop in pressure. Amplifiers have two analog outputs for pressure and pulse waveforms, and a plus gain adjustment to amplify or attenuate the pulse signal. To obtain BP, a conscious rat was placed in a restrainer with a cuff attached to a heated tail. Rats were habituated before the first measurement to enable them to adapt to the restrainer and BP measurement. In our study, we opted for the tail‐cuff method over more invasive techniques like telemetry to minimize stress and discomfort to the animals, ensuring their welfare while still obtaining reliable BP measurements. This decision aligns with ethical considerations and enhances the feasibility of conducting comprehensive cardiovascular assessments in our experimental model.

### 2.4 Urine collection

At the end of the treatment period, rats were individually placed in metabolic cages and fasted overnight for no more than 12 h (from 7 p.m to 7 a.m). The following morning, urine samples were collected, and the volume (V_urine_) was measured. The samples were then centrifuged at 1500 rpm for 5 min and stored at −80°C for further analyses.

### 2.5 Transthoracic echocardiography

At termination of the study, the rats were anesthetized using isoflurane (5% induction, and maintained at a constant concentration of 1%–2.5% delivered by 100% O_2_ mask inhalation). Subsequent to anesthesia, M‐mode echocardiographic parameters were assessed on anesthetized rats. M‐mode offers superior temporal resolution, allowing for accurate assessment of dynamic changes in cardiac structure and function during the cardiac cycle. As such, M‐mode imaging provides clear visualization of key parameters such as wall thickness, chamber dimensions, and myocardial motion, which are essential for characterizing cardiac remodeling and dysfunction (McNamara et al., 2024). A long‐axis parasternal view of the LV was obtained using a non‐invasive high‐resolution (pediatric‐10 MHz) ultrasound probe coupled to an echocardiogram (Siemens, Acuson SC2000, Diagnostic ultrasound system; Siemens Medical Solutions, USA, Inc.). LV dimensions were measured, including LV internal diameter in systole (LVIDs), left ventricular internal diameter in diastole (LVIDd), interventricular septal thickness in systole (IVSTs), interventricular septal thickness in diastole (IVSTd), LV posterior wall thickness in systole (LVPWTs), and LV posterior wall thickness in diastole (LVPWTd). Thereafter, indices of systolic function were computed using LV dimensions. First, endocardial fractional shortening (endFS) was calculated as endFS = (LVIDd − LVIDs)/LVIDd × 100. Second, midwall fractional shortening (midFS) was calculated as, midFS = [(LVIDd + PWTd) − (LVIDs + PWTs)]/[(LVIDd + PWTd)] × 100. LV relative wall thickness (RWT) was calculated as RWT = (2 × PWTd)/LVIDd. Lastly, systolic wall stress was computed as wall stress = [(SBP) × (LVIDs/2)]/2PWTs. Echocardiographic assessment was conducted by a trained sonographer who was blinded to the experimental groups. Assessments were conducted in accordance with the American Society of Echocardiography guidelines (McNamara et al., 2024).

### 2.6 Blood and tissue sampling

Following echocardiography, a thoracotomy was performed, and the rats were exsanguinated by cardiac puncture. Blood was collected into sterile EDTA tubes (plasma), centrifuged, and stored at −80°C for further analysis. The heart and kidneys were removed, weighed, and stored in an RNA stabilizing reagent, RNAlater (Ambion, Sigma Aldrich, Germany), for tissue storage to maintain the integrity of the RNA within the post‐mortem samples for reliable gene expression analysis.

### 2.7 Circulating plasma levels of inflammatory markers and urinary creatinine

Plasma concentrations of the inflammatory markers including C‐reactive protein (CRP), tumor necrosis factor alpha (TNF‐α), and pentraxin‐3 (PTX‐3), vascular cell adhesion molecule‐1 (VCAM‐1), in plasma; and creatinine (Cr) in plasma and urine were determined by solid phase sandwich enzyme‐linked immunosorbent assay (ELISA) (Elab Science Biotechnologies, Co Ltd., Wuhan, China). The lower detectable limits for TNF‐α, CRP, PTX‐3, VCAM‐1, and Cr were 78 pg/mL, 0.3 ng/mL, 0.16 ng/mL, 12.5 pg/mL, and 1.25 μg/mL, respectively. Additionally, both inter‐assay and intra‐assay coefficients of variation were below 10% for all kits used. PTX‐3 was included as a specific marker of local vascular inflammation in the context of hypertension (Adamo et al., 2020), while CRP and TNF‐α were included to determine whether local inflammatory changes summated into a systemic pro‐inflammatory state. Urine and plasma Cr concentrations were used to assess kidney function by computing the estimated glomerular filtration rate as eGFR = (Cr_urine_×V_urine_)/Cr_plasma_.

### 2.8 Total RNA extraction and cDNA synthesis

Total RNA was extracted from LV tissue using an Illustra™ RNAspin Mini Isolation kit (GE Healthcare, Buckinghamshire, United Kingdom) according to manufacturer's instructions. The presence and quantity of RNA was confirmed using a Nanodrop OneC spectrophotometer (Thermo Fisher Scientific, Waltham, USA). The RNA was then reverse‐transcribed to cDNA using SuperScript™ IV VILO™ cDNA synthesis master mix (Thermo Fisher Scientific, Life Technologies, Carlsbad, USA), according to manufacturer's instructions. The presence of cDNA was again confirmed using a Nanodrop OneC spectrophotometer (Thermo Fisher Scientific, Waltham, USA). The cDNA constructs were stored at −20°C for further analyses.

### 2.9 Relative mRNA expression of pentraxin‐3 in LV tissue

Comparative gene expression RT‐PCR was performed in duplex reactions using the cDNA templates (~1 μL), pre‐designed Taqman™ probe mixes for the reference gene Hypoxanthine Guanine Phosphoribosyl Transferase‐1 (*HPRT1*) (0.25 μL, Taqman assay ID: Rn01527838_g1, Thermo Fisher Scientific, Life Technologies, Carlsbad, USA), the gene of interest (0.5 μL, Taqman gene expression assay) and Taqman Fast Advanced PCR Master Mix (5 μL) in a final volume of 10 μL. The relative expression of the gene of interest was computed using the 2^−∆∆Ct^ method. Reporting of data complied with the conventions as set out in the Minimum Information for Publication of Quantitative Real‐Time PCR Experiments (MIQE) guidelines (Johnson et al., 2014).

### 2.10 Total collagen content from LV tissue

LV tissue samples preserved in 10% buffered formalin were processed for standard paraffin embedding. Thin sections, 5 μm in thickness, were cut from the paraffin‐embedded tissues using a microtome, followed by deparaffinization and rehydration (Mokotedi et al., 2020). The sections were then stained for 60 min at room temperature with a 0.1% sirius red solution dissolved in saturated picric acid. After staining, the slides were rinsed with acidified water, dehydrated, and mounted with Digital Picture Exchange (DPX) mounting medium. The tissue sections were examined using a Zeiss Axioscope 2 Plus microscope with a Zeiss AxioCam (Zeiss, Peabody, MA, USA, RRID:SCR_023747). Visualization was conducted under bright‐field with a 10× objective lens (oil magnification ×100). Using ImageJ software (RRID:SCR_003070), the collagen fraction area was calculated for each tissue section by dividing the collagen‐occupied area by the total tissue area (Mokotedi et al., 2020).

### 2.11 Cardiomyocyte width from LV tissue

Hematoxylin and Eosin (H&E) staining was used to measure LV cardiomyocyte width at the level of the nucleus. First, the tissue samples were deparaffinized in xylene and then rehydrated through graded ethanol concentrations. After this, the samples were stained with Harris' hematoxylin and eosin solutions, followed by dehydration in ethanol and xylene, and mounted with DPX mountant. A single evaluator who was blinded to the experimental groups examined the slides using a DVM6 digital microscope (Leica Biosystems, Vista, CA, USA), with a max. FOV of 12.55 mm and a magnification range of 46× to 675×. LV cardiomyocyte width was measured using the straight‐line tool. Each sample was measured three times, and the average width (μm) was recorded.

### 2.12 Statistical analyses

Statistical analysis was performed using GraphPad Prism version 10.0.0 for Windows (GraphPad Software, Boston, Massachusetts USA, www.graphpad.com). Normality was confirmed using a Shapiro–Wilk test. Normally distributed data is expressed as arithmetic mean ± SD. Group differences were ascertained using one‐way analysis of variance (ANOVA) test followed by a Tukey's post hoc test to correct for multiple comparisons. Associations between circulating PTX‐3 and indices of LVH were determined from linear regression analyses, with and without controlling for the confounding effect of systolic BP. Statistical significance was considered at *p* < 0.05, a priori.

## 3 RESULTS

### 3.1 Haemodynamic and gravimetric parameters

**FIGURE 2:**
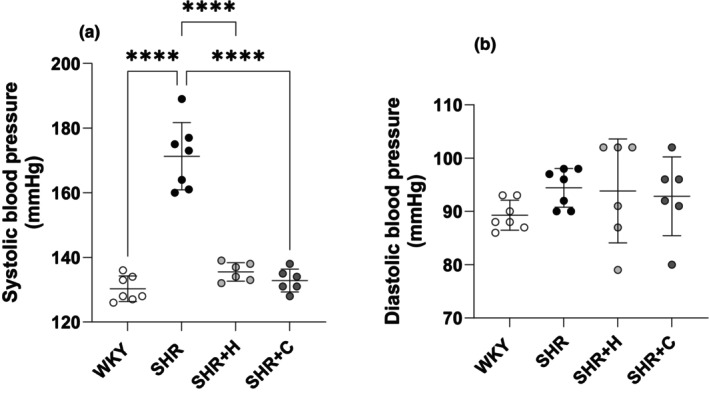
A graphical representation of the haemodynamic parameters, systolic blood pressure (a), and diastolic blood pressure (b) in normotensive WKYs, untreated SHRs, and SHRs treated with either hydralazine or captopril. ****p < 0.0001.

Untreated SHR exhibited significantly greater systolic BP compared to WKY (*p* < 0.0001). SHR treated with either hydralazine or captopril experienced a decrease in SBP by ~0.79 folds compared to untreated SHR (*p* < 0.0001). The diastolic BP was similar between all the groups (*p* > 0.05) (Figure 2a,b). WKY were significantly heavier than treated SHR (all *p* < 0.001). The hearts of untreated SHR (*p* = 0.015) and SHR treated with hydralazine (*p* = 0.040), but not captopril (*p* = 0.756), were significantly heavier compared to the hearts of WKY. The LV was significantly heavier in untreated SHR compared to WKY (*p* = 0.004). When normalized to body mass, the hearts of the three different SHR groups were significantly heavier than those of WKY (all *p* < 0.0001). SHR treated with hydralazine had significantly heavier normalized heart mass compared to SHRs treated with captopril (*p* = 0.029). When normalized to body mass, the LV of the three different SHR groups were significantly heavier than those of WKY (all *p* < 0.0001). Kidney mass (absolute and normalized to body mass) and eGFR were similar amongst all the groups (all *p* > 0.05, Table 1).

**TABLE 1:** Gravimetric analyses and kidney function in normotensive WKYs, untreated SHRs, and SHRs treated with either hydralazine or captopril.

	WKY, *n* = 7	SHR, *n* = 7	SHR + H, *n* = 6	SHR + C, *n* = 6
Body mass (g)	389 ± 15	360 ± 69	336 ± 25**	336 ± 23**
Heart mass (g)	1.28 ± 0.08	1.50 ± 0.16*	1.48 ± 0.07*	1.35 ± 0.15
Heart (g/100 g)	0.33 ± 0.02	0.42 ± 0.03***	0.44 ± 0.02***	0.40 ± 0.02^a^ ^,^ ***
LV mass (g)	0.85 ± 0.10	1.12 ± 0.17**	1.04 ± 0.09	1.01 ± 0.12
LV (g/100 g)	0.22 ± 0.03	0.31 ± 0.03***	0.31 ± 0.02***	0.30 ± 0.01***
Kidney mass (g)	1.39 ± 0.09	1.32 ± 0.16	1.27 ± 0.11	1.23 ± 0.07
Kidney (g/100 g)	0.36 ± 0.01	0.37 ± 0.04	0.38 ± 0.03	0.31 ± 0.15
eGFR (ml/min)	0.46 ± 0.21	0.29 ± 0.17	0.38 ± 0.29	0.31 ± 0.19

*Note*: Data expressed as mean ± SD. Organ masses were normalized to body mass and expressed as g/100 g. 1‐way ANOVA with Tukey post hoc test.

Abbreviations: C, captopril; eGFR, estimated glomerular filtration rate; H, hydralazine; LV, left ventricle; SHR, spontaneously hypertensive rats; WKY, Wistar Kyoto rats.

*** *p* < 0.0001.

** *p* < 0.001.

* *p* < 0.05 versus WKY.

^a^ *p* < 0.05 versus SHR + H.

### 3.2 LV geometry and systolic function

All indices of LV geometry were similar amongst the groups (all *p* > 0.05). Despite hydralazine or captopril having lowered BP in the treated SHR, the midwall fractional shortening was significantly decreased when compared to WKY (*p* = 0.043 for hydralazine) and (*p* = 0.018 for captopril). Endocardial fractional shortening and systolic wall stress were similar between the groups (all *p* > 0.05, Table 2).

**TABLE 2:** Echocardiographic parameters showing left ventricular geometry and systolic function in normotensive WKYs, untreated SHRs, and SHRs treated with either hydralazine or captopril.

	WKY, *n* = 7	SHR, *n* = 7	SHR + H, *n* = 6	SHR + C, *n* = 6
LV geometry				
LVIDs (cm)	0.35 ± 0.09	0.34 ± 0.15	0.29 ± 0.13	0.39 ± 0.18
LVIDd (cm)	0.68 ± 0.06	0.61 ± 0.08	0.67 ± 0.13	0.73 ± 0.11
IVSTs (cm)	0.38 ± 0.09	0.37 ± 0.07	0.30 ± 0.12	0.38 ± 0.05
IVSTd (cm)	0.27 ± 0.08	0.30 ± 0.09	0.23 ± 0.08	0.28 ± 0.07
LVPWTs (cm)	0.28 ± 0.09	0.34 ± 0.04	0.34 ± 0.09	0.26 ± 0.08
Systolic function				
endFS (%)	43 ± 12	45 ± 4	35 ± 13	30 ± 5
midFS (%)	30 ± 5	23 ± 3	18 ± 9*	17 ± 7*
Wall stress (kdynes.cm^−2^)	50 ± 24	57 ± 20	43 ± 31	65 ± 17

*Note*: Data expressed as mean ± SD. 1‐way ANOVA with Tukey post hoc test.

Abbreviations: C, captopril; endFS, endocardial fractional shortening; H, hydralazine; IVSTd, interventricular septal thickness in diastole; IVSTs, interventricular septal thickness in systole; LVIDd, left ventricular internal diameter in diastole; LVIDs, left ventricular internal diameter in systole; LVPWTs, left ventricular posterior wall thickness in systole; midFS, midwall fractional shortening; SHR, spontaneously hypertensive rats; WKY, Wistar Kyoto rats.

* *p* < 0.05 vs. WKY.

**FIGURE 3:**
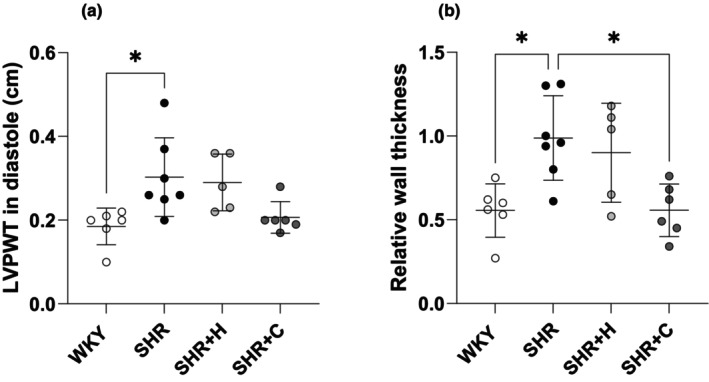
A graphical representation of indices of left ventricular hypertrophy; left ventricular posterior wall thickness in diastole (a), and relative wall thickness (b) in normotensive WKYs, untreated SHRs, and SHRs treated with either hydralazine or captopril. *p < 0.05.

Untreated SHR exhibited significantly greater LV posterior wall thickness in diastole compared to WKY (*p* = 0.023), which was neither regressed by captopril (*p* = 0.074) nor hydralazine (*p* > 0.05, Figure 3a). Relative wall thickness was greater in untreated SHR compared to WKY (*p* = 0.011) and regressed with captopril (*p* = 0.011) but not hydralazine (*p* > 0.05, Figure 3b).

### 3.3 Total collagen content and cardiomyocyte width

**FIGURE 4:**
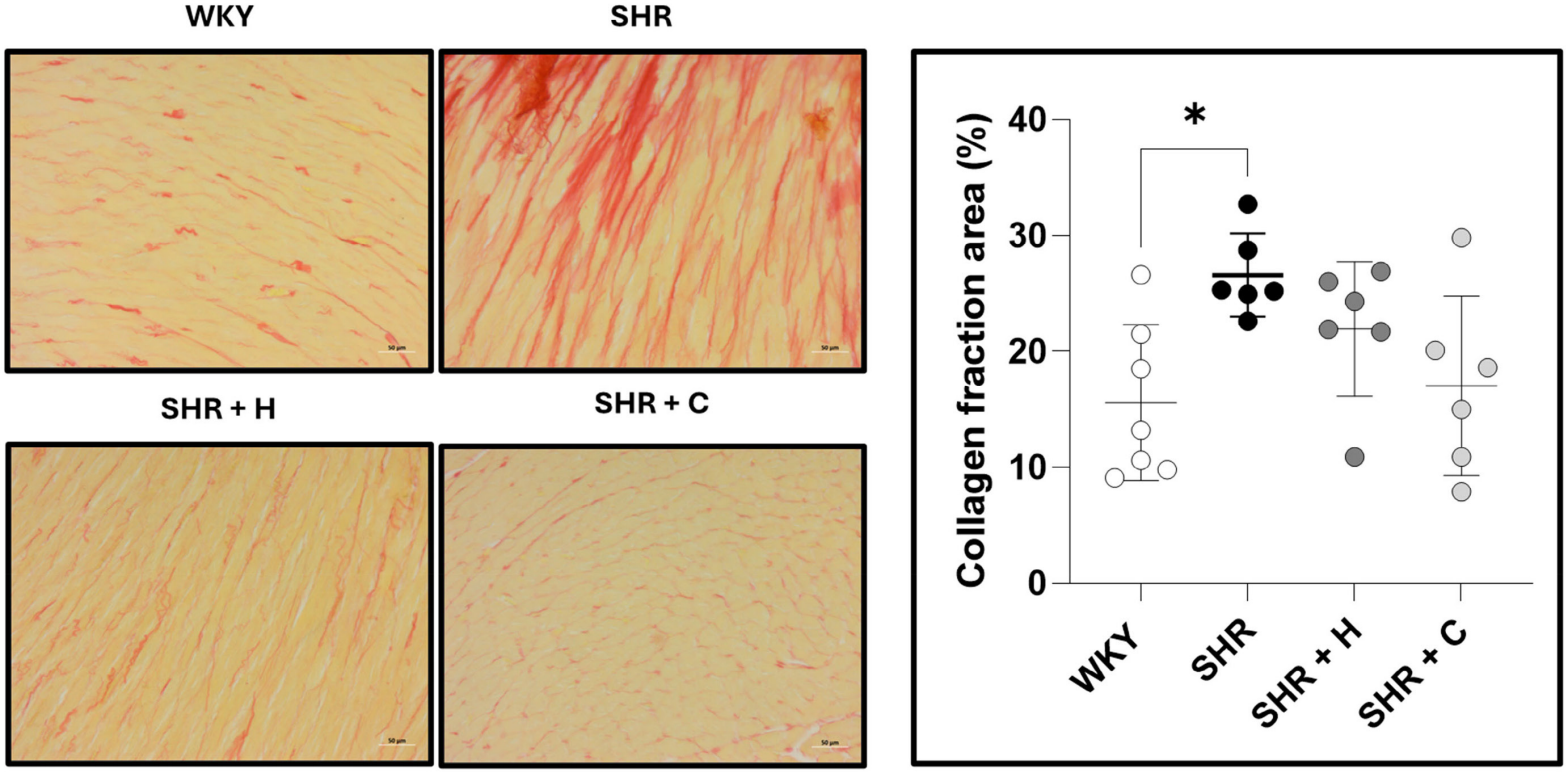
A graph depicting the collagen fraction area, expressed as a percentage of the total LV section, along with representative micrographs of normotensive WKYs, untreated SHRs, and SHRs treated with either hydralazine or captopril. *p < 0.05.

**FIGURE 5:**
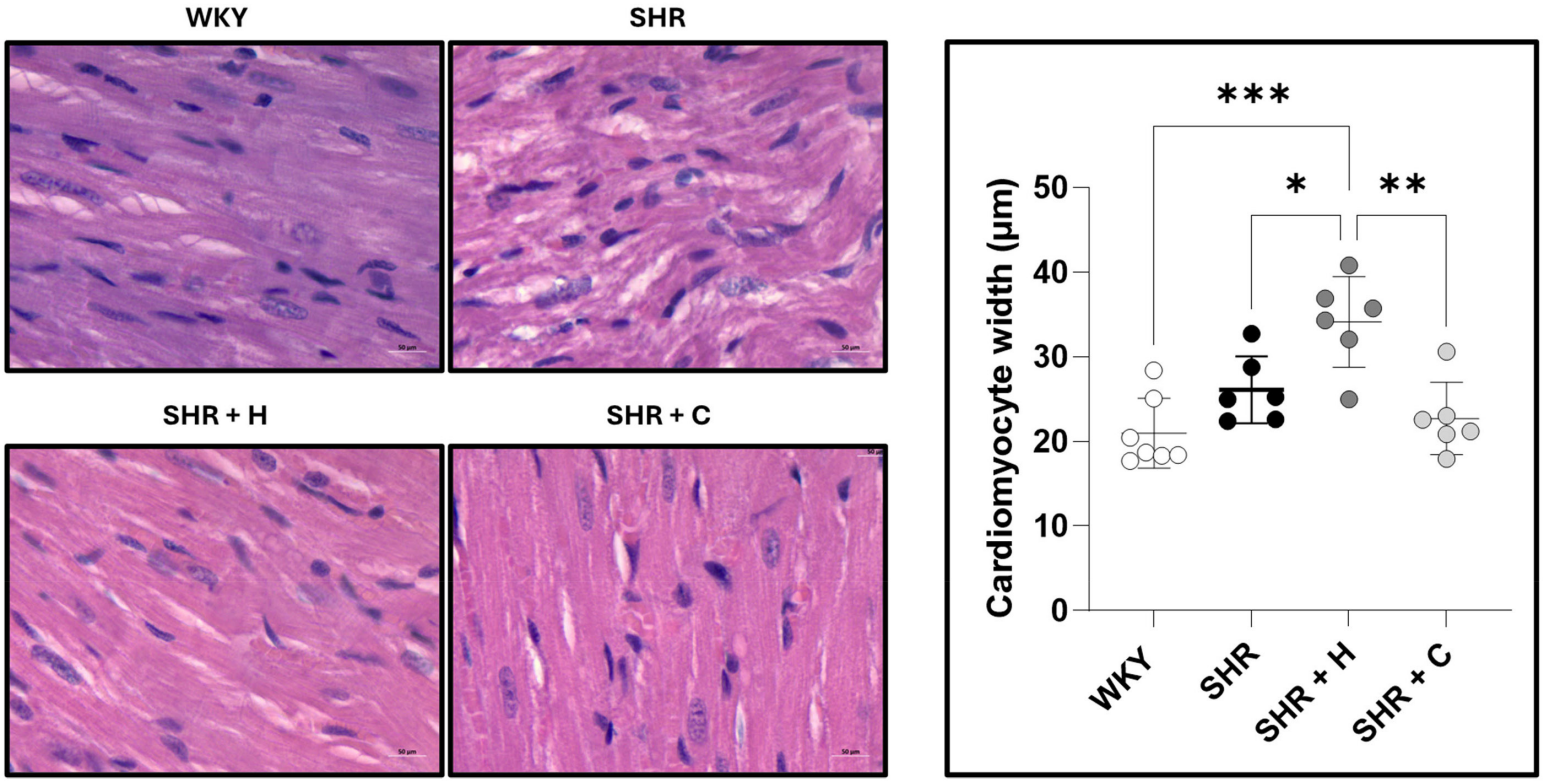
A graph depicting cardiomyocyte width, along with representative micrographs of normotensive WKYs, untreated SHRs, and SHRs treated with either hydralazine or captopril. *p < 0.05, **p < 0.001, ***p < 0.0001.

Figure 4 shows the collagen fraction area, expressed as a percentage of the total section, along with representative micrographs for each experimental group. The collagen fraction area was significantly higher in SHR compared to WKY (*p* = 0.021), but neither captopril (*p* = 0.063) nor hydralazine (*p* = 0.573) treatment reduced it. Cardiomyocyte width in SHR was similar to that of WKY (*p* = 0.197). SHR treated with hydralazine had a significantly greater cardiomyocyte width compared to WKY (*p* = 0.0002) and untreated SHR (*p* = 0.024), while captopril treatment reduced cardiomyocyte width significantly (*p* = 0.001) (Figure 5).

### 3.4 Circulating inflammatory markers

**FIGURE 6:**
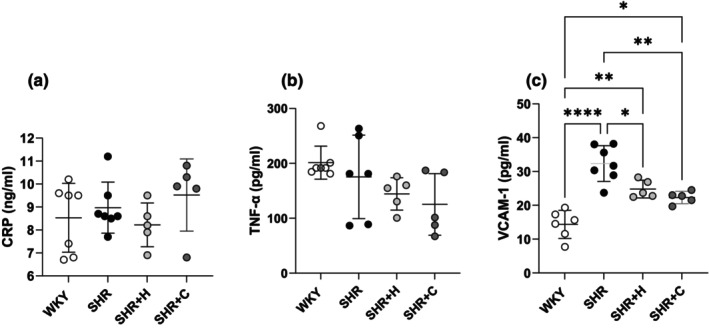
A graph illustrating the circulating concentrations of inflammatory markers including C‐reactive protein (a), tumor necrosis factor alpha (b), and vascular cell adhesion molecule‐1 (c) in normotensive WKYs, untreated SHRs, and SHRs treated with either hydralazine or captopril. *p < 0.05, **p < 0.001, ****p < 0.0001.

The circulating levels of the inflammatory markers, including CRP and TNF‐α concentrations were similar amongst the four different experimental groups (all *p* > 0.05, Figure 6a,b). Circulating concentrations of VCAM‐1 were significantly greater in SHR compared to WKY (*p* < 0.0001), and decreased significantly upon treatment with captopril (*p* = 0.002), and hydralazine (*p* = 0.019, Figure 6c).

### 3.5 Circulating pentraxin‐3 concentration and LV mRNA expression

**FIGURE 7:**
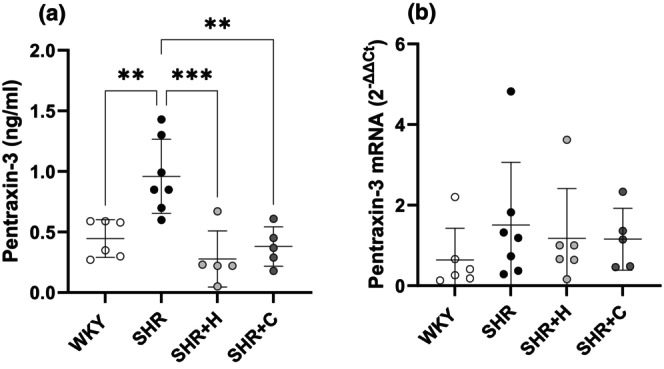
A graphical representation of circulating plasma levels of the inflammatory marker; pentraxin‐3 (a), and LV tissue‐specific mRNA expression of pentraxin‐3 (relative to HPRT1 as endogenous control), (b) in normotensive WKYs, untreated SHRs, and SHRs treated with either hydralazine or captopril. *p < 0.05, **p < 0.001, ***p < 0.0001.

Figure 7a,b show the circulating concentration of the inflammatory marker; pentraxin‐3 (Figure 7a), and LV mRNA expression of pentraxin‐3 (relative to the endogenous reference control gene; *HPRT1*, Figure 7b) in normotensive WKY, untreated SHR, and SHR treated with either hydralazine or captopril. The circulating levels of pentraxin‐3 were significantly higher in untreated SHRs compared to WKY; mean difference [95% CI], −0.51 [−0.87 to −0.15, *p* = 0.004], and were decreased by hydralazine; 0.68 [0.30–1.06, *p* = 0.0004], and captopril; 0.58 [0.20–0.96, *p* = 0.002]. However, the LV relative mRNA expression of *PTX‐3* was not different amongst the groups (all *p* > 0.05).

### 3.6 Associations between circulating levels of pentraxin‐3 with indices of left ventricular hypertrophy, inflammatory markers, kidney function, and collagen content

**FIGURE 8:**
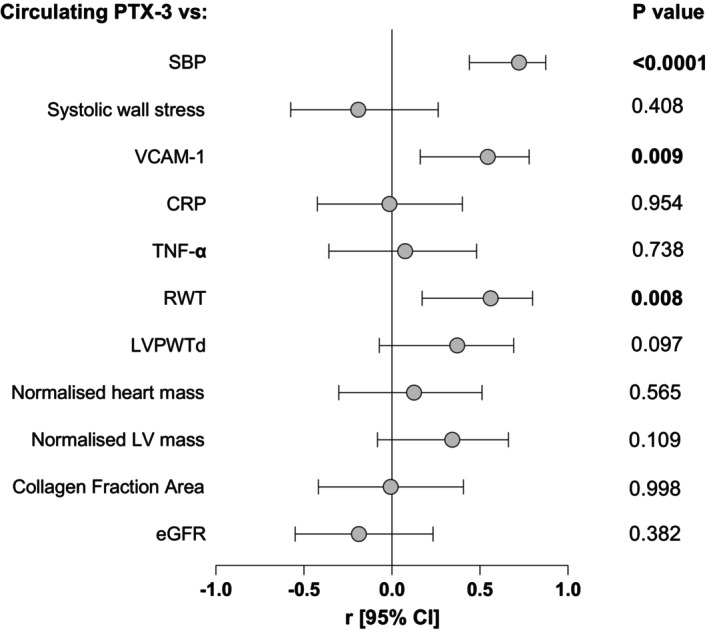
A forest plot illustrating the relationship between circulating levels of pentraxin‐3 with systolic blood pressure, indices of left ventricular hypertrophy, inflammatory markers, kidney function, and collagen fraction area in normotensive WKYs, untreated SHRs, and SHRs treated with either hydralazine or captopril. Open circles represent the point estimate (*r*, correlation coefficient). Horizontal lines bisecting the open circles represent the lower and upper limits of the 95% confidence interval (CI).

Figure 8: A forest plot illustrating the relationship between circulating concentrations of pentraxin‐3 with systolic blood pressure, indices of left ventricular hypertrophy, inflammatory markers, kidney function, and collagen fraction area in normotensive WKYs, untreated SHRs, and SHRs treated with either hydralazine or captopril. Open circles represent the point estimate (*r*, correlation coefficient). Horizontal lines bisecting the open circles represent the lower and upper limits of the 95% confidence interval (CI). Circulating concentration of pentraxin‐3 was positively and significantly associated with systolic BP (*r* = 0.722; *p* = 0.0001), VCAM‐1 (*r* = 0.545; *p* = 0.009), relative wall thickness (*r* = 0.562; *p* = 0.008), but not with systolic wall stress (*r* = −0.191; *p* = 0.408). However, in multivariable linear regression analyses adjusting for the confounding effect of SBP, relative wall thickness, *β* coefficient [95% CI]: 0.248 [−0.227–0.724, *p* = 0.286] and VCAM‐1, *β* coefficient [95% CI]: −0.004 [−0.028–0.020, *p* = 0.714] were no longer associated with circulating concentrations of pentraxin‐3. Circulating concentrations of pentraxin‐3 were also not associated with CRP (*r* = −0.013; *p* = 0.954), nor with TNF‐α (*r* = 0.076; *p* = 0.738). No associations between the circulating concentrations of pentraxin‐3 and LVPWTd (*r* = 0.372; *p* = 0.097), normalized heart mass (*r* = 0.216; *p* = 0.565) or normalized LV masses (*r* = 0.343; *p* = 0.109) were observed. Lastly, no associations were observed between pentraxin‐3 and eGFR (*r* = −0.187; *p* = 0.382) and collagen fraction area (*r* = −0.006; *p* = 0.998). Circulating concentrations of VCAM‐1 was positively and significantly associated with systolic BP (*r* = 0.755; *p* < 0.0001).

## 4 DISCUSSION

The current study shows that hypertension present in SHR subsided upon treatment with hydralazine or captopril. Furthermore, treatment with captopril but not with hydralazine resulted in the regression of concentric LVH indexed by relative wall thickness and cardiomyocyte width. We also showed that circulating PTX‐3 and VCAM‐1 concentrations were elevated with hypertension. In addition, circulating PTX‐3 and VCAM‐1 concentrations were positively associated with SBP. However, circulating PTX‐3 concentration was not independently associated with indices of concentric LVH. Finally, LV relative mRNA expression of *PTX‐3* was not different between the experimental groups. Therefore, the current study highlights the role of circulating and LV‐specific expression of PTX‐3 in the development of hypertension‐induced concentric LV remodeling.

Hypertension is one of the main risk factors for the development of concentric LVH (Lorell & Carabello, 2000). Despite some discordant results (Wu et al., 2019), circulating PTX‐3 concentration has been shown to be elevated in patients with hypertension (Carrizzo et al., 2015; Damiani et al., 2015; Yano et al., 2010; Yavuzer et al., 2016). Moreover, except in one study involving hypertensive patients with obesity (Karamfilova et al., 2022), circulating PTX‐3 concentration was associated with systolic and diastolic BP (Chen, Liu, et al., 2021; Kizilgul et al., 2017; Parlak, Aydogan, et al., 2012). Finally, anti‐hypertensive therapies, including ACE inhibitors such as enalapril, have been shown to decrease plasma levels of PTX‐3 in hypertensive patients (Buda et al., 2017; Morii et al., 2012; Parlak, Iyisoy, et al., 2012; Unlu et al., 2013). Similar to what has been shown in SHR with heart failure (Chen, Zhuang, et al., 2021), the present study showed a greater circulating PTX‐3 concentration with hypertension. In addition, the use of anti‐hypertensive therapies with different modes of action restored the circulating PTX‐3 concentration to normal levels resulting in a positive relationship between systolic BP and circulating PTX‐3 concentration.

Despite the absence of direct measurements of endothelial function, the greater circulating concentrations of VCAM‐1 in SHR as well as its association between VCAM‐1 concentrations and SBP in the present study support the role of endothelial activation in the development of hypertension (Liu et al., 1996; McCarron et al., 1994; Usui et al., 2012). Moreover, captopril is known to protect against endothelial dysfunction in humans and SHRs (Chen et al., 2008). Hydralazine, a non‐specific vasodilator, has also been shown to ameliorate endothelial dysfunction (Knowles et al., 2004). In line with the previous results, the present study showed that both captopril and hydralazine, despite having different modes of action, reduce the circulating VCAM‐1 concentrations in SHR suggesting amelioration of the endothelial dysfunction with both anti‐hypertensive therapies. PTX‐3, a pro‐inflammatory mediator released during tissue injury, may be involved in endothelial activation, an important mechanism in the establishment of hypertension (Carrizzo et al., 2015). In the present study, the concentrations of circulating PTX‐3 were associated with those of VCAM‐1. In addition, PTX‐3 was no longer associated with SBP following adjustments for VCAM‐1. Taken together, the present study suggests that PTX‐3 is involved, at least as a mediator, in response to endothelial activation and the establishment of hypertension in SHR.

While the differences in CRP or TNF‐α did not reach statistical significance, the trends in CRP or TNF‐α concentrations across groups were similar to those of PTX‐3 and VCAM‐1. Significant differences in CRP or TNF‐α concentrations may have been recorded with a larger sample size. However, a systemic pro‐inflammatory status in SHR was not confirmed in the present study, since only circulating PTX‐3 and VCAM‐1 concentrations were increased. Nonetheless, our findings confirm those of previous studies reporting that PTX‐3 is a more sensitive marker of endothelial dysfunction compared to CRP (Yano et al., 2010). Unlike CRP, which is predominantly produced in the liver, PTX‐3 is produced locally at the site of inflammation by various cell types including endothelial cells, immune cells, fibroblasts, and renal epithelial cells (Li et al., 2024; Luo et al., 2023; Nauta et al., 2005). This local production positions PTX‐3 as a more specific marker of tissue‐specific inflammatory responses compared to the systemic marker, CRP. Indeed, unlike CRP, which is produced in the liver, PTX‐3 is reported to be produced directly within the blood vessels/site of injury following endothelial activation, making it a more accurate marker of localized inflammation and tissue repair (Carrizzo et al., 2015; Kanbay et al., 2015; Knoflach et al., 2012; Kocyigit et al., 2014; Zlibut et al., 2019). However, to delineate a potential mechanism behind the differences in circulating PTX‐3, the present study lacks evidence regarding the specific source of circulating PTX‐3. Given that eGFR remained unchanged in SHR, showing no evidence of impaired kidney function, but rather suggesting an enhanced autoregulatory capacity to insulate glomeruli from barotrauma (Bidani & Griffin, 2004). For this reason, it is unlikely that circulating PTX‐3 was produced by renal epithelial cells.

As a consequence of hypertension, the heart adapts in order to neutralize the afterload by increasing the LV wall thickness (Lorell & Carabello, 2000). In our study, LV mass (absolute and normalized to body mass), LVPWTd, and RWT were greater in SHR compared to WKY, indicating presence of LVH. Variations in the degree and pattern of hypertrophy may lead to differential impacts on wall thickness and overall LV mass. Concentric hypertrophy, as indexed by increased wall thickness without proportional chamber dilation, might be more readily detected through echocardiographic parameters such as LVPWTd and RWT as compared to gravimetric assessment using organ masses. In concert with previous studies (Fouad‐Tarazi & Liebson, 1987; Levick et al., 2010; Mokotedi et al., 2019; Reddy et al., 1996), the present data demonstrate that only captopril successfully prevented the development of concentric LVH in SHR. Despite its BP‐lowering effect, hydralazine had limited beneficial effect on the development of concentric LVH in SHR. Evidence has demonstrated the differential effects of these anti‐hypertensive therapies on the development of LVH in pressure overload (Fouad‐Tarazi & Liebson, 1987). The regression of LVH by captopril may be attributed to blockade of the intra‐cardiac renin angiotensin system (Reddy et al., 1996), whereas the limited effect of hydralazine on concentric LVH may be attributed to activation of the sympathetic nervous system (Fouad‐Tarazi & Liebson, 1987; Levick et al., 2010). The concomitant activation of the sympathetic nervous system by hydralazine may condition LVH regression, albeit with opposing trophic effects that preclude regression. Therefore, the use of anti‐hypertensive drugs with differential LV hypertrophic effects may then assist in understanding the molecular pathways involved in the development of concentric LVH.

Amongst other molecular pathways, inflammation may play a key role in the development of concentric LVH (Adamo et al., 2020). PTX‐3, a mediator with chemoattractant properties released during tissue injury and repair, has been shown to be involved in the development of concentric LVH in pressure overload (Chen, Zhuang, et al., 2021; Suzuki et al., 2013). However, the role of increased physiological concentration of PTX‐3 on the development of LVH in hypertension still requires elucidation. Using the differential effects of anti‐hypertensive therapies in the present study, circulating PTX‐3 concentration was associated only with relative wall thickness and not with any other indices of concentric LVH. However, this association disappeared after adjusting for the confounding effect of SBP, suggesting that circulating PTX‐3 may not play a direct role in the development of LVH beyond its relationship with blood pressure. In this way, SBP appears to be a stronger determinant of LVH than PTX‐3, indicating that the hypertrophic changes are more likely driven by the effects of elevated blood pressure rather than a direct effect of PTX‐3. The absence of significant changes in systolic wall stress suggests that the hypertrophic response to haemodynamic pressure overload in SHR compensated sufficiently to prevent excessive wall stress. Further, systolic wall stress lacked associations with circulating concentration of PTX‐3, indicating that PTX‐3 did not modulate mechanical adaptation to pressure overload.

Some evidence shows that localized rather than systemic inflammation may contribute to the development of concentric LVH in SHR (Li et al., 2008). However, LV cardiac‐specific *PTX‐3* gene expression was not increased in SHR. The present study was performed when LVH was already established. Therefore, the present data do not unequivocally indicate that circulating or tissue PTX‐3 was not involved prior to this stage in SHR as cardiac fibroblasts may be already activated in this advanced hypertensive state. However, the observation that captopril, an anti‐hypertensive agent that decreases BP and ameliorates detrimental molecular pathways associated with hypertrophic remodeling in the myocardium, did not alter *PTX‐3* gene expression, suggests that PTX‐3 may not play a significant role in this context.

### 4.1 Limitations

The current study had limitations to consider. Although circulating plasma levels of PTX‐3 associated with SBP, causality could not be ascertained. While there was no change in the relative mRNA expression of *PTX‐3* in SHRs, which was not impacted by anti‐hypertensive treatment with either captopril or hydralazine, we did not evaluate PTX‐3 protein expression within heart tissue. It is plausible that diastolic function may be impaired. However, tissue and pulsed doppler imaging were not determined. Beyond eGFR, the present study may also benefit from extensive characterization of renal structure from histological assessment to delineate a potential mechanism behind the differences in circulating PTX‐3. Lastly, because the development of concentric LVH may be sexually dimorphic in SHR, future investigations using both sexes are warranted to further elucidate the functional role of PTX‐3 in the development of concentric LVH.

### 4.2 Conclusion

Our study highlights the involvement of PTX‐3 in the development of hypertension but not in the development of LVH using the differential effects of captopril and hydralazine in SHR. Specifically, the administration of captopril reduced systolic BP, circulating VCAM‐1 and PTX‐3 levels, as well as effectively prevented the development of concentric LVH. While hydralazine similarly decreased systolic BP and improved circulating PTX‐3 levels, it did not demonstrate any beneficial effects on the hypertrophic response. Consequently, circulating PTX‐3 concentration was positively associated with SBP but not independently associated with indices of LVH. The lack of involvement of PTX‐3 is confirmed with the similar LV relative mRNA expression of PTX‐3 between the groups despite the different phenotype related to the development of LVH. The dissociation between circulating levels of PTX‐3 and indices of concentric LVH poses significant clinical implications. Firstly, it challenges the conventional view of PTX‐3 as a biomarker directly associated with the development or progression of LVH, particularly in hypertensive individuals. Secondly, it underscores the multifactorial nature of LVH pathogenesis, which highlights the need for a more comprehensive understanding of the underlying mechanisms involved. Lastly, this suggests that PTX‐3 may not be the sole determinant of LVH in hypertensive patients and warrants further investigation into alternative biomarkers or pathways involved in the development of concentric LVH. Further investigations are warranted to elucidate the precise role of PTX‐3 in mediating adverse cardiac remodeling under conditions of pressure overload. Such future studies may provide valuable insights into the mechanistic pathways involved and aid in the development of novel therapeutic strategies aimed at mitigating hypertensive cardiac complications to broaden our understanding of the inflammatory pathways involved.

## AUTHOR CONTRIBUTIONS

**Siluleko A. Mkhize**: Conceptualization, Data curation, Formal analysis, Investigation, Methodology, Visualization, Writing—original draft, Writing—review & editing. **Ashmeetha Manilall**: Formal analysis, Supervision, Investigation, Project administration, Writing—review & editing. **Lebogang Mokotedi**: Investigation, Project administration, Writing—review & editing. **Sule Gunter**: Formal analysis, Supervision, Investigation, Project administration, Writing—review & editing. **Frederic S Michel**: Conceptualization, Data curation, Formal analysis, Investigation, Methodology, Supervision, Visualization, Writing—original draft, Writing—review & editing.

## FUNDING INFORMATION

SM received funding from the South African National Research Foundation (NRF, Reference: MND210430598430) to support his postgraduate studies enabling him to pursue this research. This work was supported by the Wits Faculty of Health Science Individual Grant (Grant holder: SM, Reference: 001.401.8521101.0000000.000000 PHSMFR0), South African NRF rated researcher competitive grant (Grant holder: FM, Reference: CSRP170502229389). The funders had no role in study design, data collection and analysis, decision to publish, or preparation of the manuscript.

## CONFLICT OF INTEREST STATEMENT

The authors declare that no conflicts of interests.

## ETHICS STATEMENT

The study was conducted in accordance with international ethical guidelines and standards for the care and use of laboratory animals, and it received approval from the Animal Research Ethics Committee of the University of the Witwatersrand (AREC clearance certificate number: 2021/03/03C). The research was carried out at the Wits Research Animal Facility, located at the University of the Witwatersrand in Johannesburg, South Africa.

## Data Availability

The data presented in this manuscript are available upon reasonable request to the corresponding author.
